# Polypoid Adenomyoma of the Uterus

**DOI:** 10.7759/cureus.4044

**Published:** 2019-02-11

**Authors:** Nida Sajjad, Hina Iqbal, Kumail Khandwala, Shaista Afzal

**Affiliations:** 1 Radiology, Aga Khan University Hospital, Karachi, PAK

**Keywords:** mesenchymal tumor, atypical polypoid adenomyoma, uterus

## Abstract

Polypoid adenomyoma is a rare uterine endometrial polypoid tumor of mixed epithelial and mesenchymal origin. Although the clinical and pathologic features of polypoid adenomyomas have been described extensively, imaging findings for these tumors have not been frequently reported in the literature. On imaging, their features may be confused with prolapsed leiomyomas or malignancy. Hemorrhagic cystic spaces in a prolapsed uterine tumor within the vagina should raise consideration of a diagnosis of polypoid adenomyoma. Such blood-containing cystic spaces would be unusual findings in leiomyomas and malignancy. Diagnosing polypoid adenomyoma is vital because it can potentially be managed by hysteroscopic resection, unlike an ordinary form of adenomyosis.

## Introduction

Polypoid adenomyoma of the uterus is an endometrial polyp in which the stromal component is made up of smooth muscle [1]. These are benign tumors and account for 1.3% of all endometrial polyps. Polypoid adenomyomas are of mixed epithelial and mesenchymal origin [2]. Although their clinical and pathological features have been described well in literature, imaging findings for these tumors have been seldom reported.

We report a case of a 44-year-old woman with urinary retention who had a prolapsed polypoidal uterine lesion on imaging which was confirmed to be polypoid adenomyoma on histopathology. We aim to review the imaging findings and the relevant literature on this rare entity.

## Case presentation

**Figure 1:**
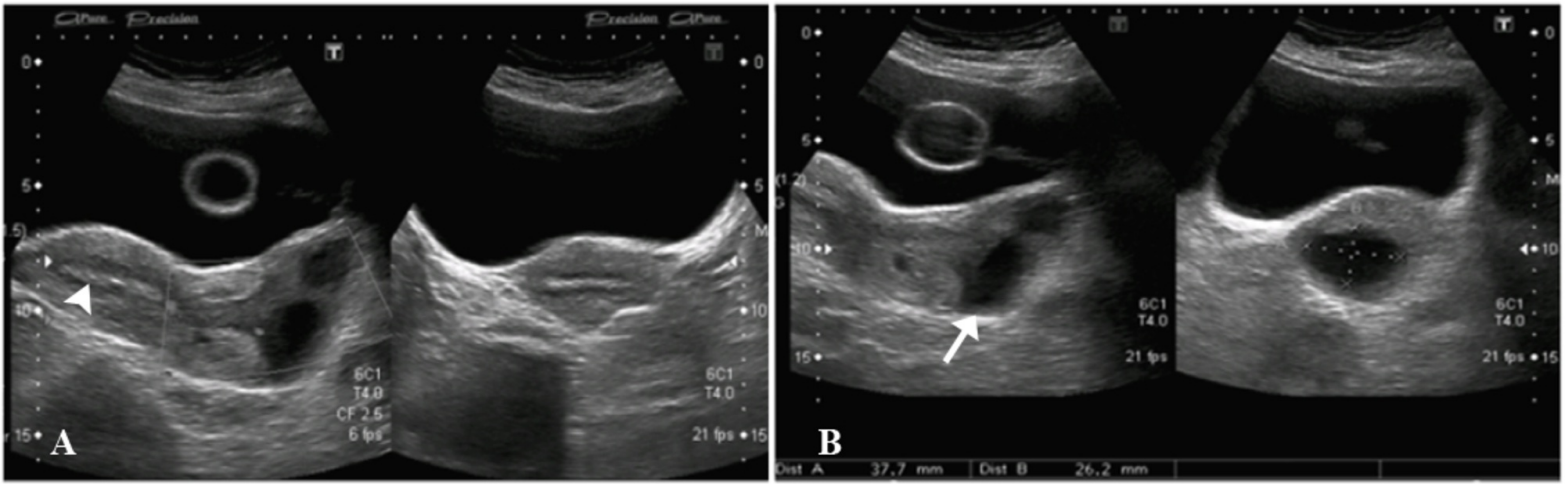
Transabdominal ultrasound images. A) Uterine fundal endometrium appears normal (arrowhead). B) Heterogenous area (arrow) in the superior one-third of vagina with two anechoic cystic spaces within it containing internal echoes. Foley's catheter balloon noted within the urinary bladder.

**Figure 2:**
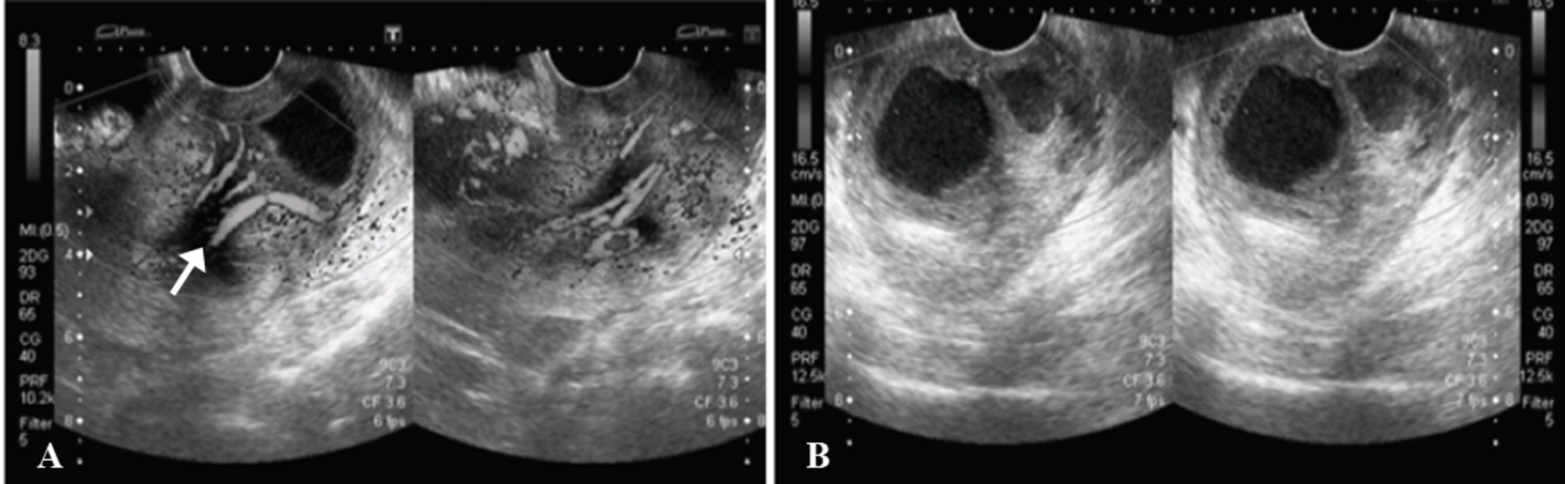
Transvaginal ultrasound images. A) Significant vascularity (arrow) noted on color Doppler in the lower uterine endometrium and cervical canal which was extending into the heterogenous area within the upper vagina. B) Predominantly anechoic cystic lesions are redemonstrated in the vagina, containing fine internal echoes.

A 44-year-old previously healthy woman presented to the emergency department complaining of urinary retention. Transabdominal (Figure 1) and transvaginal (Figure 2) ultrasound showed a heterogeneous area measuring 53 x 27 mm in the superior one-third of vagina with evidence of cystic spaces containing internal echoes. Significant vascularity was seen in the lower endometrium and cervix which was extending into this heterogeneous area. The sonographic findings were concluded as a pedunculated endometrial polyp or prolapsed fibroid with cystic degeneration.

**Figure 3:**
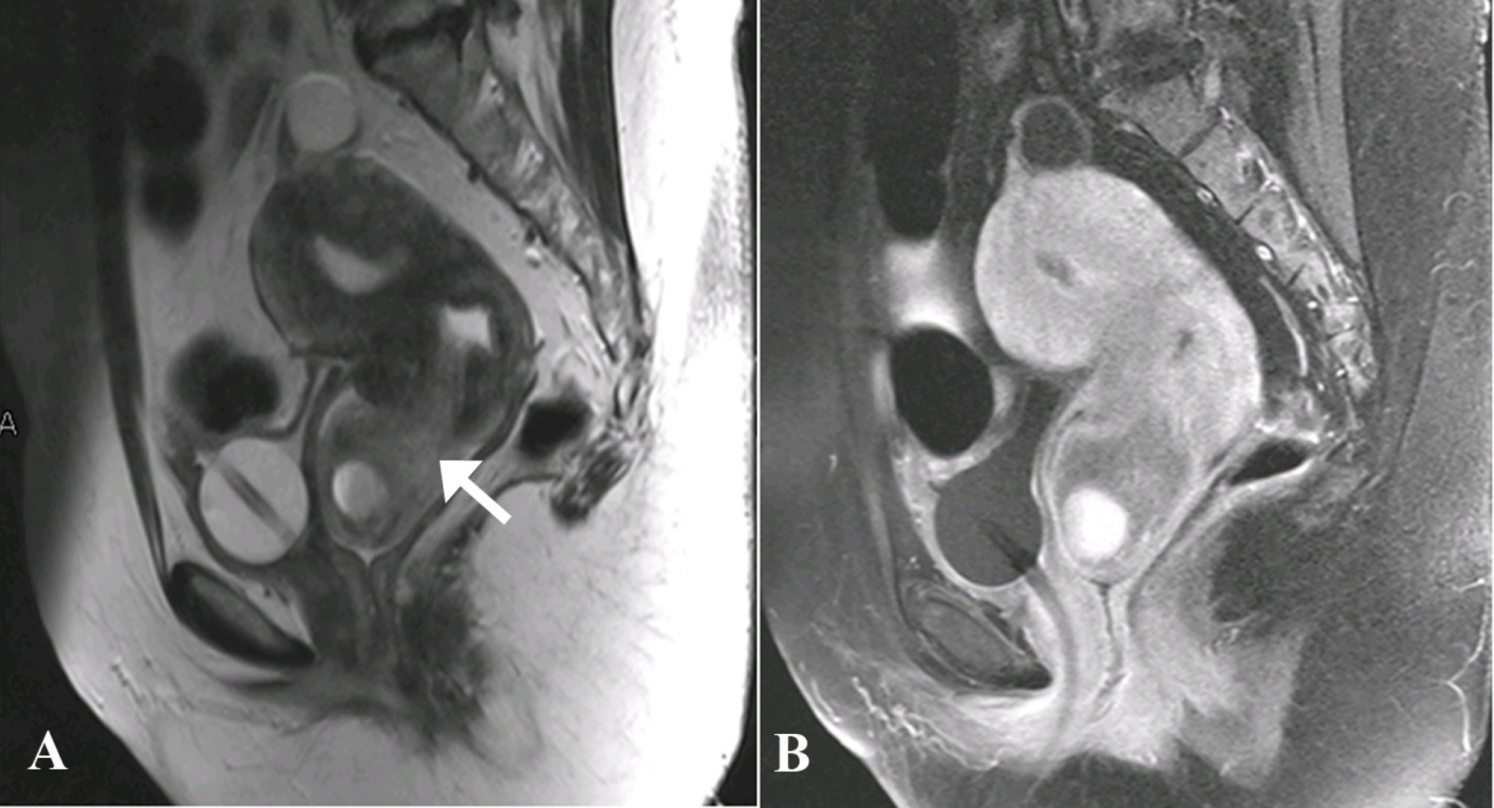
Magnetic resonance images - A) Sagittal T2-weighted and B) Sagittal T1-post contrast. A) A large, well-defined, abnormal signal intensity polypoidal mass lesion (arrow) distending the endocervical canal and extending through the external os lying in the upper one-third of vagina. Cystic spaces noted within the polypoidal lesion in the vagina which are showing fluid-fluid level suggesting hemorrhage. Junctional zone is also ill-defined and diffusely thickened in the uterus measuring approximately 12 mm suggesting adenomyosis. B) The lesion is enhancing heterogeneously. Post-contrast enhancement is relatively less than that of myometrium.

**Figure 4:**
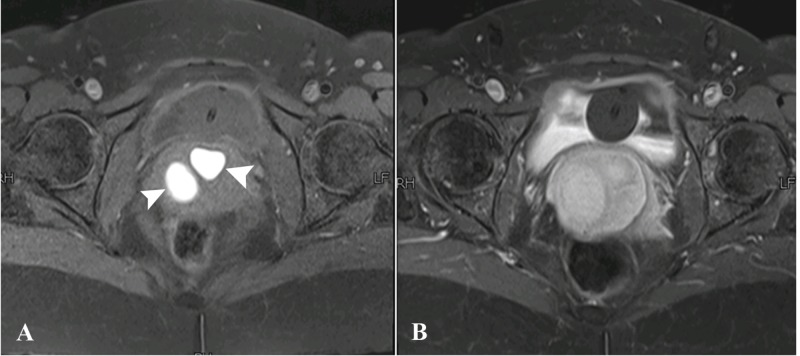
Magnetic resonance images - A) Axial pre- and B) Axial post-contrast T1-fat-sat sequences. A) Sections at the level of upper third of vagina showing rounded T1-hyperintense structures (arrowheads) representing hemorrhagic cystic spaces. B) These structures were not showing fat suppression.

**Figure 5:**
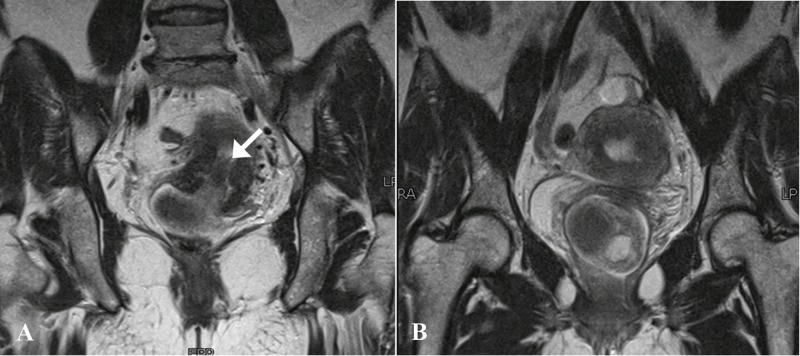
Magnetic resonance images - Coronal T2-weighted sequences. A) A T2-hypointense stalk was seen extending inferiorly from the endometrium and cervix (arrow). B) The stalk was connected to the polypoidal lesion with cystic spaces appreciated within the vagina.

Magnetic resonance imaging (MRI) of the pelvis with contrast was then done which demonstrated a large, well-defined abnormal signal intensity polypoidal mass distending the endocervical canal and extending through the external os (external orifice) into the upper one-third of the vagina. It was measuring 46 x 46 x 58 mm in maximum dimensions. It appeared to be connected to the uterine endometrium by a T2-hypointense stalk seen within the endometrial cavity. No evidence of invasion into adjacent structures was seen. The uterine junctional zone was also thickened and ill-defined, which was suggestive of adenomyosis. The lesion contained rounded T1-hyperintense cystic spaces with fluid-fluid level within it, suggestive of hemorrhages. The lesion did not show significant diffusion restriction, and enhanced heterogeneously in the post-contrast study. Post-contrast enhancement was relatively less than that of myometrium (Figures 3-5).

The initial differential diagnoses included endometrial polyp or submucosal leiomyoma with cystic degeneration. Prolapsed malignancy was a less likely differential because there was no diffusion restriction. The patient proceeded to have a hysteroscopy, endometrial biopsy and vaginal myomectomy. Histopathology of the specimen revealed polypoid adenomyoma, with no evidence of malignancy.

## Discussion

An adenomyoma, which protrudes into the endometrial cavity, is referred to as a polypoid adenomyoma [3]. These tumors are rare and can be divided into typical and atypical types based on the histopathology. Histologically, typical polypoid adenomyomas have regular, benign, tubular endometrial glands within a benign appearing smooth muscle stroma and well-demarcated myometrial smooth muscle [4]. On the other hand, endometrial glands with varying degrees of atypia within a myofibromatous stroma are identified in atypical polypoid adenomas [5]. Grossly polypoid adenomyomas appear no different than ordinary endometrial polyps. The presenting symptom is usually abnormal vaginal bleeding and generally occurs in patients of the reproductive age group [6]. Our patient presented unusually with urinary retention, possibly due to mass effect of the lesion in the vagina.

On ultrasound, polypoid adenomyomas can appear as solid, well-circumscribed endometrial masses with cystic areas [2]. On MRI, they are found to be well-defined intracavitary uterine masses and may involve the lower uterine segment, endocervix or uterine corpus. On T1-weighted images they are usually isointense. Signal intensity on T2-weighted images may depend on the size and amount of glands within the tumor. The presence of a visible stalk passing through the cervix and connecting a prolapsed mass back to the uterus is a helpful imaging finding that is often best appreciated at MRI and indicates a prolapsed uterine tumor, as seen in our patient. The stalk connecting an apparent cervical mass to the endometrial cavity, seen on MR imaging, is referred to the broccoli sign [7]. Some of these tumors may also show hemorrhage within the cystic spaces, which appear as hyperintense foci on T1-weighted images and are not suppressed on fat-suppressed T1-weighted imaging [8].

Therefore, hemorrhagic cystic spaces in a prolapsed uterine tumor are suggestive of a diagnosis of typical polypoid adenomyoma, especially in a premenopausal female with additional findings suggestive of adenomyosis as seen in our case. Blood-containing cystic spaces would be infrequently seen in leiomyomas and malignancy, although potentially can be seen in the setting of leiomyomas with red degeneration and in uterine sarcomas [9]. The enhancement pattern of polypoid adenomyomas is said to be irregular/heterogeneous. Diagnosing polypoid adenomyoma is essential because it may be managed by hysteroscopic resection, unlike an ordinary form of adenomyosis [10].

## Conclusions

Although the clinical and pathological features of polypoid adenomyomas have been described extensively, imaging findings for these tumors have been infrequently reported in the literature. Hemorrhagic cystic spaces in a prolapsed uterine tumor within the vagina should raise consideration of a diagnosis of polypoid adenomyoma. Such blood-containing cystic spaces would be unusual findings in leiomyomas and malignancy.
